# Supplementary material for: Schistosomiasis outbreak during COVID-19 pandemic in Takum, Northeast Nigeria: Analysis of infection status and associated risk factors
Source: PLoS One. 2022 Jan 21;17(1):e0262524. doi: 10.1371/journal.pone.0262524 (PMC8782311; doi:10.1371/journal.pone.0262524)
Supplement: S1 Appendix — (DOCX) [file pone.0262524.s001.docx]

**Questionnaire**

*Guide: The questionnaire is to be administered by a trained interviewer to the study participant (if the study participant is a minor, a parent or legal guardian must be present at the point of interview).*

**Referencing details:**

**Country: Region: State: Community: Household number:**

**-----------------------------------------------------------------------------------------------------**

1. **Demographic details**
2. Name of Respondent: ………………………..................
3. Gender.......
4. Age: ……….
5. Participant Identification Number:

**-----------------------------------------------------------------------------------------------------**

1. **Water, sanitation and hygiene (WASH) facilities**

**1. Type of water sourc**e (a) Public tap (b) Stream/river (c) Hand pump borehole

(d) Protected dug well (e) Unprotected dug well

(f) Rain water (g) others (specify)

**2. Types of toilet facility** (a) Flush Toilet (b) Ventilated Improved pit

(c) Pit latrine without slab (d) Pit latrine with slab

(e) None/Open defecation in nearby bush (f) Others

3. **Handwashing facilities in toilets (**a) yes (b) no

4. **If yes, what are the handwashing facilities** (a) water (b) water and soap (c) ash (d) none

………………………………………………………………………………………………….

**(c) Water contact practices**

***Guide: please tick the water contact practices the participants has exhibited in the last 3 months.***

1. **Bathing in the stream or river (**a) yes (b) no
2. **Washing of clothes in the stream or river (**a) yes (b) no
3. **Fishing from the stream or river (**a) yes (b) no
4. **Fetching water from the stream or river (**a) yes (b) no
5. **Playing in the stream or river (**a) yes (b) no
6. **Swimming in the stream or river (**a) yes (b) no

………………………………………………………………………………………………….

**Parasitological examinations**

**Participant Identification Number**: ………

**Laboratory results**

| **SCH** | Number of egg/10 ml of urine |
| --- | --- |
| *S. haematobium* |  |
| *S. mansoni* |  |

**Name of Technologist: …………………… Signature and Date:………………………**
