# Supplementary material for: Schistosomiasis outbreak during COVID-19 pandemic in Takum, Northeast Nigeria: Analysis of infection status and associated risk factors
Source: PLoS One. 2022 Jan 21;17(1):e0262524. doi: 10.1371/journal.pone.0262524 (PMC8782311; doi:10.1371/journal.pone.0262524)
Supplement: S2 Appendix — (DOCX) [file pone.0262524.s002.docx]

**Tambaya**

***Jagora: Tambayar da za a ba da ita ita ce mai ba da horo mai horarwa ga mahalarcin binciken (idan ɗan takarar ƙarami ne, dole ne iyaye ko mai kula da doka su kasance a wurin yin hira).***

**Ƙarin bayani:**

**Ƙasa: Yanki: Jiha: Al'umma: Lambar gida:**

**-----------------------------------------------------------------------------------------------------**

1. **Bayanin alƙaluma**

1. Sunan wanda ake kara: ………………………..................

2. Jinsi.......

3. Shekaru:.………..

4. Lambar Shaidar Mahalarta:

1. **Ruwa, tsaftar muhalli da tsaftar muhalli (WASH)**

1. Nau'in tushen ruwa (a) Matsa jama'a (b) Ruwa/kogi (c) Rijiyar burtsatse

(d) Rijiyar da aka kare ta kariya (e) Rijiyar da ba ta da kariya

(f) Ruwan sama (g) wasu (saka)

2. Nau'o'in kayan bayan gida (a) Ruwan bayan gida (b) Ingantaccen rami

(c) Makamin rami ba tare da slab ba (d) Ramin bango da slab

(e) Babu/Buɗe bayan gida a cikin daji kusa (f) Wasu

3. Kayan wanke hannu a bayan gida (a) na’am (b) a'a

4. Idan eh, menene wuraren wanke hannu (a) ruwa (b) ruwa da sabulu (c) toka (d) babu

…………………………………………………………………………………………………

1. **Ayyukan tuntuɓar ruwa**

***Jagora: da fatan za a yi ishara da hanyoyin tuntuɓar ruwa waɗanda mahalarta suka nuna a cikin watanni uku da suka gabata.***

1. Yin wanka a rafi ko kogi (a) na’am (b) a'a
2. Wanke tufafi a cikin rafi ko kogi (a) na’am (b) a'a
3. Kifi daga rafi ko kogi (a) na’am (b) a'a
4. Samun ruwa daga rafi ko kogi (a) na’am (b) a'a
5. Yin wasa a cikin rafi ko kogi (a) na’am (b) a'a
6. Yin iyo a rafi ko kogi (a) na’am (b) a'a

**……………………………………………………………………………………………………….**

1. **Nazarin Parasitological**

Lambar Shaidar Mahalarta: .........

Sakamakon dakin gwaje -gwaje

| **SCH** | Yawan ƙwai/10 ml na fitsari |
| --- | --- |
| *S. haematobium* |  |
| *S. mansoni* |  |

**Masanin fasaha: …………………… Sa hannu da Rana:………………………**
