# Supplementary material for: Schistosomiasis outbreak during COVID-19 pandemic in Takum, Northeast Nigeria: Analysis of infection status and associated risk factors
Source: PLoS One. 2022 Jan 21;17(1):e0262524. doi: 10.1371/journal.pone.0262524 (PMC8782311; doi:10.1371/journal.pone.0262524)

PLOS ONE

Schistosomiasis outbreak during COVID-19 pandemic in Takum, Northwest Nigeria: Analysis of Infection status and associated risk factors

--Manuscript Draft--

**Manuscript Number:**	PONE-D-21-30831
**Article Type:**	Research Article
**Full Title:**	Schistosomiasis outbreak during COVID-19 pandemic in Takum, Northwest Nigeria: Analysis of Infection status and associated risk factors
**Short Title:**	Factors associated with schistosomiasis outbreak in Takum, Northwest Nigeria
**Corresponding Author:**	Hammed O. Mogaji, B.Sc. M.Sc Ph.D<br>Federal University Oye-Ekiti<br>Ekiti, Ekiti NIGERIA
**Keywords:**	Schistosomiasis; Hematuria; Outbreak; Elimination; Nigeria; Takum; Taraba
**Abstract:**	Background<br><br>Mass drug administration for schistosomiasis started in 2014 across Taraba State. Surprisingly in 2020, an outbreak of haematuria was reported in Takum local government area. This epidemiological investigation therefore assessed the current status of infection, analysed associated risk factors and arrested the outbreak through community sensitization activities and mass treatment of 3,580 persons.<br><br>Methods<br><br>Epidemiological assessment involving parasitological analysis of stool and urine samples were conducted among 432 consenting participants in five communities. Samples were processed using Kato-Katz and urine filtration techniques. Participants data on demography, water contact behaviour and access to water, sanitation and hygiene facilities were obtained using standardized questionnaires. Data were analysed using SPSS 20.0 and significance level was set at 95%.<br><br>Results<br><br>An overall prevalence of 34.7% was observed, with 150 participants infected with both species of Schistosoma parasite. By communities, prevalence was higher in Birama (57.7%), Barkin Lissa (50.5%) and Shibong (33.3%). By species', S. haematobium infection was significantly higher than S. mansoni (28.9% vs 9.5%), with higher proportion of younger males infected ($p<0.05$). The condition of WASH is deplorable. About 87% had no latrines, 67% had no access to improved source of potable water and 23.6% relied on the river as their main source of water. Infections was significantly associated with water contact behaviours like playing in water (OR:1.50, 95% CI: 1.01-2.25) and swimming (OR:1.55, 95% CI: 1.04-2.31).<br><br>Conclusion<br><br>It is important to reclassify the treatment needs of Takum LGA based on the findings of this study. Furthermore, efforts targeted at improving access to WASH, reducing snail population, improving health education and strengthening surveillance systems to identify schistosomiasis hotspots will be a step in the right direction
**Order of Authors:**	Francisca Olamiju
	Hammed O. Mogaji, B.Sc. M.Sc Ph.D
	Obiageli Nebe
	Perpetua Amedu-Agbi
	Rita Urude
	Apake Elizabeth

Powered by Editorial Manager® and ProduXion Manager® from Aries Systems Corporation

	Olatunwa Olamiju
	Chimdinma Okoronkwo
	Ayodele Marcus
	Ijeoma Achu
	Okezie Mpama
**Additional Information:**	
**Question**	**Response**
**Financial Disclosure**<br><br>Enter a financial disclosure statement that describes the sources of funding for the work included in this submission. Review the submission guidelines for detailed requirements. View published research articles from *PLOS ONE* for specific examples.<br><br>This statement is required for submission and **will appear in the published article** if the submission is accepted. Please make sure it is accurate.<br><br>**Unfunded studies**<br>Enter: *The author(s) received no specific funding for this work.*<br><br>**Funded studies**<br>Enter a statement with the following details:<br>• Initials of the authors who received each award<br>• Grant numbers awarded to each author<br>• The full name of each funder<br>• URL of each funder website<br>• Did the sponsors or funders play any role in the study design, data collection and analysis, decision to publish, or preparation of the manuscript?<br>• **NO** - Include this sentence at the end of your statement: *The funders had no role in study design, data collection and analysis, decision to publish, or preparation of the manuscript.*<br>• **YES** - Specify the role(s) played.<br><br>* typeset	The author(s) received no specific funding for this work.
**Competing Interests**<br><br>Use the instructions below to enter a competing interest statement for this submission. On behalf of all authors,	The authors have declared that no competing interests exist.

disclose any competing interests that could be perceived to bias this work—acknowledging all financial support and any other relevant financial or non-financial competing interests.<br><br>This statement is required for submission and **will appear in the published article** if the submission is accepted. Please make sure it is accurate and that any funding sources listed in your Funding Information later in the submission form are also declared in your Financial Disclosure statement.<br><br>View published research articles from *PLOS ONE* for specific examples.<br><br>**NO authors have competing interests**<br><br>Enter: *The authors have declared that no competing interests exist.*<br><br>**Authors with competing interests**<br><br>Enter competing interest details beginning with this statement:<br><br>*I have read the journal's policy and the authors of this manuscript have the following competing interests: [insert competing interests here]*<br><br>* typeset	
**Ethics Statement**<br><br>Enter an ethics statement for this submission. This statement is required if the study involved:<br><br>• Human participants<br>• Human specimens or tissue<br>• Vertebrate animals or cephalopods<br>• Vertebrate embryos or tissues<br>• Field research<br><br>Write "N/A" if the submission does not require an ethics statement.	Ethical clearance for this study was obtained from the Taraba State Ministry of Health ethics review board. A pre-survey contact/advocacy meeting was made to each selected study community to obtain consent from community leaders and other major stakeholders after explaining the objectives of the research to them. This was followed by community mobilization and sensitization using town announcers to communicate the objectives of our visit to community members. Sensitization was done in all religious and public places like schools and market squares to promote participation. Community members willing to participate in the study completed written consent forms on the day of sample collection. Assent forms was completed in cases where the willing member is below 16 years of age. In this case, parents or any legal guardian were asked to accompany minors under age 16 to the sample collection site, to provide additional consents. The method of consent assertion was through thumbprint on already printed informed consent forms (ICFs).

General guidance is provided below. Consult the submission guidelines for detailed instructions. **Make sure that all information entered here is included in the Methods section of the manuscript.**<br><br>**Format for specific study types**<br><br>**Human Subject Research (involving human participants and/or tissue)**<br>• Give the name of the institutional review board or ethics committee that approved the study<br>• Include the approval number and/or a statement indicating approval of this research<br>• Indicate the form of consent obtained (written/oral) or the reason that consent was not obtained (e.g. the data were analyzed anonymously)<br><br>**Animal Research (involving vertebrate animals, embryos or tissues)**<br>• Provide the name of the Institutional Animal Care and Use Committee (IACUC) or other relevant ethics board that reviewed the study protocol, and indicate whether they approved this research or granted a formal waiver of ethical approval<br>• Include an approval number if one was obtained<br>• If the study involved *non-human primates*, add *additional details* about animal welfare and steps taken to ameliorate suffering<br>• If anesthesia, euthanasia, or any kind of animal sacrifice is part of the study, include briefly which substances and/or methods were applied<br><br>**Field Research**<br><br>Include the following details if this study involves the collection of plant, animal, or other materials from a natural setting:<br>• Field permit number<br>• Name of the institution or relevant body that granted permission	
**Data Availability**<br><br>Authors are required to make all data	No - some restrictions will apply

Powered by Editorial Manager® and ProduXion Manager® from Aries Systems Corporation

underlying the findings described fully available, without restriction, and from the time of publication. PLOS allows rare exceptions to address legal and ethical concerns. See the PLOS Data Policy and FAQ for detailed information.<br><br>A Data Availability Statement describing where the data can be found is required at submission. Your answers to this question constitute the Data Availability Statement and **will be published in the article**, if accepted.<br><br>**Important:** Stating 'data available on request from the author' is not sufficient. If your data are only available upon request, select 'No' for the first question and explain your exceptional situation in the text box.<br><br>Do the authors confirm that all data underlying the findings described in their manuscript are fully available without restriction?	
**Describe where the data may be found in full sentences. If you are copying our sample text, replace any instances of XXX with the appropriate details.**<br><br>• If the data are **held or will be held in a public repository**, include URLs, accession numbers or DOIs. If this information will only be available after acceptance, indicate this by ticking the box below. For example: *All XXX files are available from the XXX database (accession number(s) XXX, XXX.*).<br>• If the data are all contained **within the manuscript and/or Supporting Information files**, enter the following: *All relevant data are within the manuscript and its Supporting Information files.*<br>• If neither of these applies but you are able to provide **details of access elsewhere**, with or without limitations, please do so. For example:<br><br>*Data cannot be shared publicly because of [XXX]. Data are available from the*	The data underlying this study have been presented to ESPEN as part of a nationwide data. Any other request can be shared with the corresponding author.

XXX Institutional Data Access / Ethics Committee (contact via XXX) for researchers who meet the criteria for access to confidential data.<br><br>*The data underlying the results presented in the study are available from (include the name of the third party and contact information or URL).*<br>• This text is appropriate if the data are owned by a third party and authors do not have permission to share the data.<br><br>* typeset	
Additional data availability information:	

Manuscript Click here to access/download;Manuscript;Article file.docx

Schistosomiasis outbreak during COVID-19 pandemic in Takum, Northwest Nigeria: Analysis of Infection status and associated risk factors

Francisca Olamiju[1], Obiageli Nebe[2], Hammed Mogaji[,3], Perpetua Amedu-Agbi[2], Rita Urude[2], Apake Elizabeth[1], Olatunwa Olamiju[1], Chimdinma Okoronkwo[1], Ayodele Marcus[1], Ijeoma Achu[1], Okezie Mpama[1]

[1]Mission To Save The Helpless (MITOSATH), Jos, Nigeria; [2]Neglected Tropical Disease Unit, Federal Ministry of Health, Nigeria; [3]Parasitology and Epidemiology Unit, Department of Animal and Environmental Biology, Federal University Oye-Ekiti

Corresponding author: Mogaji, Hammed Oladeji[1*], Department of Animal and Environmental Biology, Federal University Oye-Ekiti, Nigeria, *mogajihammed@gmail.com

Abstract

Background

Mass drug administration for schistosomiasis started in 2014 across Taraba State. Surprisingly in 2020, an outbreak of [illegible]maturia was reported in Takum local government area. This epidemiological investigation therefore assessed the current status of infection, analysed associated risk factors and arrested the outbreak through community sensitization activities and mass treatment of 3,580 persons.

Methods

Epidemiological assessment involving parasitological analysis of stool and urine samples were conducted among 432 consenting participants in five communities. Samples were processed using Kato-Katz and urine filtration techniques. Participants data on demography, water contact behaviour and access to water, sanitation and hygiene facilities were obtained using standardized questionnaires. Data were analysed using SPSS 20.0 and significance level was set at 95%.

Results

An overall prevalence of 34.7% was observed, with 150 participants infected with both species of Schistosoma parasite. By communities, prevalence was higher in Birama (57.7%), Barkin Lissa (50.5%) and Shibong (33.3%). By species', *S. haematobium* infection was significantly higher than *S. mansoni* (28.9% vs 9.5%), with higher proportion of younger males infected ($p<0.05$). The condition of WASH is deplorable. About 87% had no latrines, 67% had no access to improved source of potable water and 23.6% relied on the river as their main source of water. Infections was significantly

associated with water contact behaviours like playing in water (OR:1.50, 95% CI: 1.01-2.25) and swimming (OR:1.55, 95% CI: 1.04-2.31).

Conclusion

It is important to reclassify the treatment needs of Takum LGA based on the findings of this study. Furthermore, efforts targeted at improving access to WASH, reducing snail population, improving health education and strengthening surveillance systems to identify schistosomiasis hotspots will be a step in the right direction

Keywords: Schistosomiasis, [illegible]aturia, Outbreak, Elimination, Nigeria, Takum, Taraba

Introduction

Schistosomiasis is an acute and chronic parasitic disease, caused by a water-borne trematode of the genus Schistosoma. Owing to the burden associated with this disease, the World Health Organization (WHO) classified it as one of the major and most common neglected tropical diseases (NTDs) requiring public health attention [1] Schistosomiasis is as well a focal disease [2], with a wide geographic distribution [3,4]. Currently, over 206 million people in 78 countries are affected with about 24,000 deaths and 2.5 million disability-adjusted life years (DALYs) [3]. The disease thrives in tropical and subtropical regions, especially among rural and marginalized urban populations without access to water, sanitation and hygiene (WASH) facilities [1,4-6].

It is estimated that at least 90% of those affected and requiring treatment for schistosomiasis live in Africa [4]. In this region, there are two major species of Schistosoma; the first is the *S. haematobium* which inhabits the vesicular and pelvic venous plexus of the bladder and causes urogenital schistosomiasis and the second is *S. mansoni* which is more often in the inferior mesenteric veins draining the large intestine and causes intestinal schistosomiasis [4,6]. In addition, the former has been reported in the Middle East and Corsica, while the latter has a wider distribution in the Middle East, the Caribbean, Brazil, Venezuela and Suriname [4]. The pathologies associated with both species vary depending on factors not limited to the severity of infection, migration of the worms through the organs and body tissues and inflammatory responses to the presence of the eggs laid by the adults [4,7,8]. Intestinal schistosomiasis can result in symptoms such as abdominal pain, diarrhoea, blood in the stool, and in more severe cases, enlargement of the liver and spleen, a condition known as hepatosplenomegaly [4,9].

However, haematuria, which is classified as the passage of v[illegible]ble or invisible blood in urine is a common symptom of urogenital schistosomiasis [4]. Other complicated pathologies may include fibrosis of the bladder and ureter, kidney damage and in more advanced cases cancer of the bladder [4]. Urogenital schistosomiasis may become more complex in females in a condition known as female genital schistosomiasis (FGS), which may present with symptoms such as genital lesions, vaginal bleeding, pain during sexual intercourse and infertility [10-12]. The pathologies are worsened among children because of the developing immunity, with already established evidence on anemia, stunting, protein-energy malnutrition, school absenteeism and reduced cognition [13-15].

Children below age 1[illegible]main the most vulnerable and represent the target group for most control interventions [5]. Ongoing elimination effort involves mass drug administration (MDA) of praziquantel to school-aged children between age 5 and 14 years in endemic areas following already established guidelines [5]. Since 2010, the WHO has coordinated the annual distribution of 250 million praziquantel donated by Merck and co. to several endemic countries with about 95.3 million people treated in 2019 [3]. Nigeria is one of the schistosomiasis endemic countries in Africa [1]. Of the 774 government areas ([illegible]As), about 708 LGAs had been mapped by the Federal Ministry of Health (FMoH), with 608 of them being endemic [16]. Treatment commenced since 2009 in Taraba and other 26 states with the support of WHO, UNICEF and partner organizations such as Mission to save the helpless (MITOSATH), Sightsavers, AMEN foundation among others [16]. Taraba is one of the 36 states in Nigeria, located in the northeastern region. Th[illegible]ate has 16 administrative units, referred to as local government areas (LGAs). All the LGAs were mapped for schistosomiasis in 2010 and subsequently in 2014 by the FMoH and other supporting partners [16,17]. During the mapping phase, a total of 80

schools was surveyed (5 schools per LGAs) with urine and stool examination from 3,936 school-aged children [17]. Takum was one of the LGAs mapped, with a low prevalence of 4% across the five schools examined in Sufa, Gboko, Kwambai, Taku[illegible]nd Takum communities. The LGA was then classified to be of low endemicity, and benefitted from biennial treatment strategy targeted at school-aged children since 2014 [17]. The therapeutic coverage in this LGA was optimal in the last 5 rounds of mass drug administration (MDA). In August 2020, during the COVID-19 pandemic, an o[illegible]eak of hematuria was reported in both children and adults across eleven communities in Takum (Barki Lissan, Liji, Takpa, Shibong Igbang, Lukpo, Kashimbila, Birama, Bibbi, Bawuro, Gamga and Mamga) (Fig 1). These communities were not part of the communities mapped in 2014, which calls for urgent public health action. The study was [illegible]ducted therefore conducted to (1) re-assess the prevalence of schistosomiasis in these communities; (2) document the status of water, sanitation and hygiene (WASH) resources; (3) identify risk factors promoting the transmission of schistosomiasis (4) treat the entire population and create awareness about schistosomiasis and (5) provide recommendations to improve program planning and implementation. In this paper, we, therefore, summarize the findings from the epidemiological study conducted and the programmatic actions implemented in line with the global target of eliminating schistosomiasis.

Methodology

Ethical statement and considerations

Ethical clearance for this study was obtained from the Taraba State Ministry of Health ethics review board. A pre-survey contact/advocacy meeting was made to each selected study community to obtain consent from community leaders and other major stakeholders after explaining the objectives of the research to them. This was followed by community mobilization and sensitization using town announcers to communicate the objectives of our visit to community members. Sensitization was done in all religious and public places like schools and market squares to promote participation. Community members willing to participate in the study completed written consent forms on the day of sample collection. Assent forms was completed in cases where the willing member is below 16 years of age. In this case, parents or any legal guardian were asked to accompany minors under age 16 to the sample collection site, to provide additional consents. The method of consent assertion was through thumbprint on already printed informed consent forms (ICFs).

Study area

This study was carried out in five communities located in Takum LGA, Taraba state, Northeastern, Nigeria. Takum is one of the 16 LGAs in Taraba state, with an approximate land area of 2,503 km^2 (Figure 2)[illegible]he climate of the area is tropical with vegetation characterized by a typical Guinea savannah interspersed with gallery forest. The annual rainfall ranges between 1,200mm and 2,000mm annually, while the average temperature is between 28 and 32°C reaching a peak at 37 °C in March and April. In addition, the area has several ponds, streams and rivers, which provides conducive environment for farming

and fishing occupation, as well as sites for other recreational activities such as bathing, swimming, and washing of clothes.

Study design and selection of communities

This study employed a cross-sectional sampling design involving questionnaire administration and sample collection in five communities out of the 11 communities that reported hematuria outbreak in the LGA. These communities (Barkin lissa, Birama, Gamga, Shibong and Takpa) were randomly selected using a balloting method. After selection, the severity of the outbreak reported to the district health officer was re-examined, and compared among selected schools and those that were not selected. Replacements were done where necessary to ensure a balance of priority. Preliminary contacts with the sub-district coordinator NTD unit, serving each ward were made in advance, before visiting the communities. The epidemiological study which was conducted in September, 2020, involved 4 distinct phases; (1) advocacy and sensitization; (2) questionnaire administration; (3) sample collection and laboratory examination and (4) treatment of all persons.

Sample size determination and selection of study participants

This study employed a total sampling methodology, following the method previously described by [18]. In brief, considering the fact that the study aimed to investigate the factors associated with the outbreak, all members of the community were mobilized and invited to participate in the study. Mobilization occurred using town announcers, and a sampling spot, at the center of the community was given to the research team for field

process. This area has a secluded space for administering the questionnaire and sorting of samples before transportation to the laboratory.

Questionnaire administration

In this study, four separate data collection forms were used. The first, which is the demographic form was used to capture the name, sex and age of participants. The form was also used to assign a unique identification number. The details on this form were used to recruit the participant into the study after completing the consent form. Participants unique identification number was used to allocate a pre-labeled sterile stool and urine specimen bottle. Furthermore, the WASH form was used to capture information about participants' access to water, sanitation and hygiene facilities. This was also accompanied by a water contact practice form, which was used to document the range of water contact activities the participants performed in the last three months. The last form is the laboratory assessment form, which was used to document the findings from the laboratory assessment of the urine and stool specimen. Before data collection, research assistants were trained on how to capture data electronically using Kobo collect tool and LINKS system on smartphones. All data were collected electronically and transferred to a remote backup server immediately after each interview. All interviews were held in confidence in a private space, except when the interviewee is a minor and needs the assistance of a legal guardian or a parent.

Collection of stool and urine samples

Participants were provided with two sterile specimens bottle, pre-labeled with their unique identification number, an applicator stick, a plain sheet of paper and a tissue paper to clean their anus. Participants were instructed to defecate on the plain sheet of paper and

use the applicator stick to transfer a fresh portion into the first bottle. Furthermore, they were instructed to provide approximately 10ml of urine in the second bottle. Samples bottled were retrieved within 1 hour of distribution, and all study participants were provided with soap and water to wash their hands appropriately. In addition, the children were provided incentives like [illegible]cuits, while the adults were provided with bar soap. Participants were also treated with 400mg/kg of praziquantel.

Parasitological assessment of stool and urine samples

All collected stool and urine samples were sorted and transported for processing within 2hours of collection to the Parasitology laboratory located in Takum General Hospital. The urine filtration method was employed to identify *S. haematobium* eggs. In brief, 10ml of urine sample was vigorously shaken and passed through a Nytrel filter with a 40 µm mesh size. The filter was then placed on a clean microscopic slide and viewed under the microscope using the x10 and x40 objective lens in search of an egg with a characteristic terminal spine. For each slide, the fields were re-examined and eggs were re-counted by another microscopist for quality assurance. Similarly, stool specimens were processed using the Kato-Katz technique. Two thick smears were prepared from a single stool sample and allowed to clear for 30 minutes before microscopic examination for *S. mansoni*. The fields were also re-examined and counter-check by another microscopist. For both urine and stool specimens, a participant is considered i[illegible]ted, if there is an egg count recorded on both sheets of the two microscopists who examined the smears.

Treatment of all consenting persons and sensitization about schistosomiasis

Following field procedures, the NTD unit at the sub-district level performed a door to door administration of praziquantel (400mg/kg) to [illegible]persons in the community. During

their visits, they sensitized the household members about schistosomiasis and the need to avoid contact with the river. They also emphasized prompt reporting of symptoms such as bloody urine to the nearest health center. The field team was supervised by a team comprising the NTD coordinators from the FMoH, the state and the LGA.

Data management and analysis

Data obtained were downloaded from the remote server by the biostatistician, and imported into Microsoft Excel for sorting before analysis in SPSS 20.0 software. Descriptive statistics including frequencies and percentages were used to describe the variables, while Chi-square statistics and logistics regression was used to estimate association and the magnitude of association between the prevalence data and other variables. A significant level was set at 95%.

Result

Demographic characteristics of study participants

A total of 432 community residents from five communities; Barkin lissa (97, 22.5%), Birama (71, 16.4%), Gamga (76, 17.6%), Shibong (96, 22.2%) and Takpa (92, 21.3%) were enrolled into this study. The majority of the participants were males (218, 50.5%), compared to females (214, 49.5%), and there was a significant difference in the gender distribution across the communities (p=0.00). By age category, the majority of the participants were between age 5 and 10 (152, 35.2%), followed by those above 21 years (130, 30.1%), 11-16 years (112, 25.9%) and 17-20 years (38, 8.8%). There were also significant differences between the age category of participants across the study communities (p=0.02). (Table 1)

Prevalence of schistosomiasis among the study participants

Of the 432 participants examined, a total of 150 (34.7%) were infected with both species of Schistosoma parasite; 125 (28.9%) for *S. haematobium*, and 41 (9.5%) for *S. mansoni*. Prevalence level varies across the communities, with the highest recorded in Birama (57.7%), followed by Barkin Lissa (50.5%), Shibong (33.3%), Takpa (17.4%) and Gamga (15.8%). (Table 2). Prevalence was higher among males and children below age 16 (Figure 3, 4). By species' prevalence, *S. haematobium* infection was significantly higher among males ($P<0.05$), but there was no significant difference in the proportion of males or females infected with *S. mansoni* ($p>0.05$) (Figure 3).

Access to water, sanitation and hygiene (WASH) facilities and prevalence of schistosomiasis

Table 3 shows the status of water supply, sanitation and hygiene (WASH) facilities. The majority of the study participants (288, 66.7%) had no regular source of potable water supply, while a high percentage of them relied on the river as their main source of water supply (102, 23.6%). Only 6.5% of the participants had access to the handpump borehole. Furthermore, the majority of the participants had no latrines (375, 86.8%) and over 40% of them had no handwashing facilities. Of all the WASH variables examined, only access to river was significantly associated with reduced odds of infection (OR:0.27; 95% CI: 0.1-0.66).

Water contact behavior among the study participants.

Out of the six water contact practices investigated, fishing (43, 10%) was the least common practice in the study areas. However, the majority of the participants engage in activities such as bathing (419, 97%), washing of clothes (357, 82.6%), fetching water (358, 82.9%), playing in river (193, 44.7%) and swimming (214, 49.5%). Infections were significantly associated with playing and swimming activities with increased odds of 1.50 (95% CI: 1.01-2.25) and 1.55 (95% CI: 1.04-2.31), respectively. (Table 4).

Treatment data

A total 3,580 person were treated across the study communities. More persons were treated in Shibong (n=1,057), followed by Birama (n=1,044), Barki Lisa (n=634), Gamga (n=632) and Takpa (n=213). Furthermore, treated males (n=1912) were more than treated females (n=1668), and treated persons above aged 15 (n=2,436) were more than school-aged children between age 5 and 14 (n=1124).

Discussion

The outbreak of urogenital schistosomiasis was unexpected in Takum LGA, considering the optimal records of therapeutic coverage for praziquantel MDA since 2014. Due to the pandemic, the response to this outbreak involved a lot of processes, which made it more challenging since MDA had ceased, with schools closed and restrictions to public gatherings and movements. Nevertheless, the epidemiological team responded swiftly and arrested the outbreak through mass treatment of all individuals above age 5 in concordance with the standard operating guidelines stipulated for resuming MDA amid COVID-19 pandemic. It is therefore necessary to present the learnings from the epidemiological analysis of the outbreak, more importantly, the current status of infection, associated risk factors and recommendations to forestall future occurrence.

The prevalence reported in this study corroborates with the hematuria outbreak, with two of the communities having an overall prevalence above 50%, another had a prevalence above 30% and two communities had their prevalence between 16 and 17%. The moderate prevalence (<50%) recorded in the other three communities could be attributed to the fact that targeted administration of Praziquantel was carried out before the arrival of the epidemiological team. Also, on an aggregated basis, the pattern of infection across the communities might have been masked, since the prevalence across these five communities is 34.7%. This aggregation could misinform program actions targeted at eliminating the disease [18]. Until now, Takum was classified to be a low endemic LGA since 2014 and had been receiving biennial treatment [17]. This outbreak and our prevalence reports, therefore, highlight the need to re-classify the LGA for annual treatment, and also support the ongoing discussion on precision mapping and disaggregation of data during planning and implementation of MDA [2]. This becomes

very important considering the focality of schistosomiasis, and the complex life cycle involving a mixture of human behavior and availability of snail intermediate host in conducive water bodies.

WASH has been advocated severally as a complementary tool to ongoing MDA programme focused on schistosomiasis[19-21]. Surprisingly, the odds of infection reduced among those who regard the river as their source of drinking water. The collection of water for drinking has been reported as a relatively less important pathway of infection because it involves immersion of small areas of the body and for relatively short durations unlike other activities like bathing, swimming or playing [20,22]. To support this submission, our results show that other water contact activities such as playing and swimming, which would require more contact time with the river were significantly associated with increased odds of infection, with those who visit the river to swim or play been twice more exposed than those who do not. This finding conforms with earlier reports of [20].

Swimming and playing are risk factors that are common among male young school-aged children]23,24]. Our findings also support this as the majority of those infected participants in our study were young male children below age 15. It is possible that the primary source of this outbreak might be from a segment of these young population who got in contact with the water body via swimming or playing, urinated in the process around the peak periods, thus supporting the transmission of schistosomiasis. This thought is in line with a similar outbreak reported in Zimbabwe [25]. This segment of the population might have been heavily infected and under-treated because of the previous misclassification of the LGA on treatment basis. On the other hand, the closure of

schools during the pandemic era supports clustering and more contact hours between young school-aged children at river sites, from different communities and could also be another pathway of contamination of river sites [26].

Notwithstanding, the epidemiological risk analysis has raised the following substantial concerns that could have supported the outbreak; (i) lack of baseline mapping in the study communities which calls for more refined approaches such as precision mapping, (2) misclassification of the LGA based on treatment needs which resulted in undertreatment (3) predominant risky behavior of swimming and playing among the young children which might have been compounded by the lockdown imposed from the pandemic, and (4) availability of a pool of viable intermediate snail host at the river sites. These concerns, therefore, reflect gaps that need to be addressed in line with the goal of eliminating schistosomiasis by 2030.

It is therefore imperative to consider; (1) investments in effort targeted at reclassifying the LGA and adjusting the treatment thresholds (2) strengthening surveillance system to identify hot-spots such as areas with high reportage of hematuria, (3) investments in the epidemiological mapping of infections when resources allow, (4) continuous sensitization of young children, most especially as schools have resumed on the dangers of excessive recreational activities at the river site is important, and (5) investments in efforts targeted at reducing the snail population in the river body associated with these communities.

Conclusion

Until now, Takum was classified to be a low endemic LGA and had been receiving biennial treatment. This outbreak and our prevalence reports highlight the need to re-classify the LGA and adjust the treatment thresholds. In addition, our findings support the ongoing discussion on precision mapping and disaggregation of data during planning and

implementation. Swimming and playing in rivers were the most potent risk factor supporting the transmission of schistosomiasis. Strengthening available surveillance systems to identify hotspots and investments in efforts targeted at improving health education of children and reducing snail population will be a step in the right direction

Limitation of the study

Although participation was voluntary, some participants may be afraid to join the study because of their perception of the pandemic. As such, we cannot ignore the impact the COVID-19 pandemic had on participant recruitment which might have affected our sample size in each of the communities.

List of abbreviations

FMoH: Federal Ministry of Health

FGS: Female Genital Schistosomiasis

GSA: Global Schistosomiasis Alliance

LGAs: Local Government Areas

MDA: Mass Drug Administration

NTD: Neglected Tropical Diseases

SPSS: Statistical Package for Social Sciences

SCI: Schistosomiasis Control Initiative

WHO: World Health Organization

Declarations

Ethical approval and consent to participate

Ethical clearance for this study was obtained from the Taraba State Ministry of Health ethics review board. A pre-survey contact/advocacy meeting was made to each selected study community to obtain consent from community leaders and other major stakeholders after explaining the objectives of the research to them. This was followed by community mobilization and sensitization using town announcers to communicate the objectives of our visit to community members. Sensitization was done in all religious and public places like schools and market squares to promote participation. Community members willing to participate in the study completed written consent forms on the day of sample collection. Assent forms was completed in cases where the willing member is below 16 years of age. In this case, parents or any legal guardian were asked to accompany minors under age 16 to the sample collection site, to provide additional consents. The method of consent assertion was through thumbprint on already printed informed consent forms (ICFs).

Consent for publication

Not applicable

Availability of data and materials

The datasets used and/or analyzed during the current study are available from the corresponding author on reasonable request.

Competing interests

The authors declare that they have no competing interest

Funding

Not applicable

Authors' contribution

FO, OO, ON and AE conceptualized the study. ON, PA, RU prepared the protocol, while IA and OM improved the protocol. PA, RU, IA, OM participated in field surveys involving data collection and laboratory analysis of specimens. FO, OO, ON and AE provide supervised the field surveys. HM, AM and CO performed all statistical analysis and HM prepared the first draft of the manuscript. All authors contributed to the development of the final manuscript and approved its submission.

Acknowledgements

We are grateful to the community leaders across the study areas, and all the health workers who gave sacrificed their time during the outbreak despite the pandemic situation.

References

1. Hotez PJ, Kamath A. Neglected Tropical Diseases in Sub-Saharan Africa: Review of Their Prevalence, Distribution, and Disease Burden. PLoS Negl Trop Dis. 2009; 3(8): e412.

2. Global Schistosomiasis Alliance (GSA). Accelerating Progress for Schistosomiasis Conttrol and Elimination Post-2020. https://www.eliminateschisto.org/sites/gsa/files/content/attachments/2019-06-26/Final_GSA_Accelerating%20Progress%20For%20Schistosomiasis%20Control%20and%20Elimination%20Post%202020%20Meeting%20Report_0.pdf

3. World Health Organization (WHO). Ending the neglect to attain the Sustainable Development Goals: a road map for neglected tropical diseases 2021–2030. Geneva: World Health Organization; 2020. Licence: CC BY-NC-SA 3.0 IGO.

4. World Health Organization (WHO). Schistosomiasis. 2021. https://www.who.int/news-room/fact-sheets/detail/schistosomiasis. Accessed 20 September 2021

5. World Health Organization (WHO). Helminth control in school-age children: a guide for managers of control programmes. 2021. https://www.who.int/neglected_diseases/resources/9789241548267/en/. Accessed 20 September 2021

6. Midzi N, Mduluza T, Chimbar MJ, Tshuma C, Charimar L, et al. Distribution of schistosomiasis and soil transmitted helminthiasis in Zimbabwe: towards a national plan of action for control and elimination. PLoS Negl Trop Dis. 2014;8, e3014.

7. Centre for Disease Control (CDC). Schistosomiasis. Laboratory identification of parasites of public health concern. https://www.cdc.gov/dpdx/schistosomiasis/index.html. Accessed 20 September 2021

8. Costain AH, MacDonald AS, Smits HH. Corrigendum: Schistosome Egg Migration: Mechanisms, Pathogenesis and Host Immune Responses. Front. Immunol. 2019;10: 749. https://doi.org/10.3389/fimmu.2019.00749

9. Jose RL. Acute schistosomiasis mansoni: revisited and reconsidered. Mem Inst Oswaldo Cruz, Rio de Janeiro. 2010;105(4):422-435.doi.org/10.1590/S0074-02762010000400012

10. Kjetland EF, Leutscher PDC, Ndhlovu PD. A review of female genital schistosomiasis. Trends in Parasitol. 2012:28:58–65.

11. Kjetland EF, Norseth HM, Taylor M, Lillebo K, Kleppa E et al. Classification of the lesions observed in female genital schistosomiasis. Int. J. Gynecol. Obstet. 2014:127: 227–228.

12. Ekpo UF, Odeyemi OM, Sam-wobo SO, Onunkwor OB, Mogaji HO et al. Female genital schistosomiasis (FGS) in Ogun State, Nigeria: a pilot survey on genital symptoms and clinical findings. Parasitology Open, 2017;3(10):1-9. doi:10.1017/pao.2017.11

13. Bustinduy AL, Parraga IM, Thomas CL, Mungai PL, Mutuku F, et al. Impact of polyparasitic infections on anemia and undernutrition among Kenyan children living in a Schistosoma haematobium-endemic area. Am J Trop Med Hyg. 2013;88(3):433–40. pmid:23324217;

14. Guerrant RL, Leite AM, Pinkerton R, Medeiros PH, Cavalcante PA et al. Biomarkers of Environmental Enteropathy, Inflammation, Stunting, and Impaired Growth in Children in Northeast Brazil. PLoS ONE. 2016;11(9):e0158772. pmid:27690129; PubMed Central PMCID: PMCPMC5045163.

15. Osakunor DNM, Woolhouse MEJ, Mutapi F. Paediatric schistosomiasis: What we know and what we need to know. PLoS Negl Trop Dis. 2018;12(2): e0006144. https://doi.org/10.1371/journal.pntd.0006144

16. Federal Ministry of Health (FMOH), Neglected Tropical Diseasea Master Plan 2015-2020. https://espen.afro.who.int/system/files/content/resources/NIGERIA_NTD_Master_Plan_2015_2020.pdf

17. Mission to Save the Helpless (MITOSATH). Epidemiological Mapping survey report in Taraba State, Nigeria. Unpublished Data.

18. Mogaji HO, Dedeke GA, Bada BS, Bankole S, Adeniji A et al. (2020) Distribution of ascariasis, trichuriasis and hookworm infections in Ogun State, Southwestern Nigeria. PLoS ONE. 2020;15(6): e0233423. https://doi.org/10.1371/journalpone.0233423

19. Freeman MC, Ogden S, Jacobson J, Abbott D, Addiss DG et al. Integration of water, sanitation, and hygiene for the prevention and control of neglected tropical diseases: a rationale for inter-sectoral collaboration. PLoS Neg Trop Dis. 2013;7(9), e2439. https://doi.org/10.1371/journal.pntd.0002439

20. Grimes JE, Croll D, Harrison WE, Utzinger J, Freeman MC et al. The roles of water, sanitation and hygiene in reducing schistosomiasis: a review. Parasit Vectors. 2015:**8:**156. https://doi.org/10.1186/s13071-015-0766-9

21. Campbell SJ, Savage GB, Gray DJ, Atkinson J-AM, Soares Magalhães RJ et al. Water, Sanitation, and Hygiene (WASH): A Critical Component for Sustainable Soil-Transmitted Helminth and Schistosomiasis Control. PLoS Negl Trop Dis. 2014;8(4): e2651. https://doi.org/10.1371/journal.pntd.0002651

22. Tayo MA, Pugh RN, Bradley AK. Malumfashi Endemic Diseases Research Project, XI. Water-contact activities in the schistosomiasis study area. Ann Trop Med Parasitol. 1980;74:347–54.

23. Sady H, Al-Mekhlafi HM, Mahdy MAK, Lim YAL, Mahmud R, et al. Prevalence and Associated Factors of Schistosomiasis among Children in Yemen: Implications for an Effective Control Programme. PLoS Negl Trop Dis. 2013;7 (8): e2377. https://doi.org/10.1371/journal.pntd.0002377

24. Deribe K, Eldaw A, Hadziabduli S, Kailie E, Omer MD, et al. High prevalence of urinary schistosomiasis in two communities in South Darfur: implication for interventions. Parasit Vectors: 2011;4: 14.

25. Chimberengwa PT, Masuka N, Gombe NT, Bangure D, Tshimanga M et al Schistosomiasis outbreak investigation, Empandeni Ward, Mangwe District, Matabeleland South Province, June 2012; a case control study. BMC Res Notes. 2014;7:623. https://doi.org/10.1186/1756-0500-7-623

26. Schistosomiasis Control Initiative (SCI) Foundation. Treating schistosomiasis and parasitic worm infections in Malawi during COVID-19. https://schistosomiasiscontrolinitiative.org/news/2020/8/11/treating-parasitic-worm-infections-in-malawi-during-covid-19

Figure legend

Figure 1: Samples of bloody urine collected during study procedures

Source: The authors took this picture on using their camera with the permission of the participants.

Permission: The authors give permission to re-use this image.

Figure 2: Map of Taraba State showing the study LGA

Source: The authors using their primary data in ArcGIS software created this map.

Permission: The authors give permission to re-use this map.

Figure 3: Prevalence of schistosomiasis by sex among the study participants

Source: The authors using their primary data to create this chart in Microsoft Excel software.

Permission: The authors give permission to re-use this map.

Figure 4: Prevalence of schistosomiasis by sex among the study participants

Source: The authors using their primary data to create this chart in Microsoft Excel software.

Permission: The authors give permission to re-use this map.

Figure 1

Click here to access/download;Figure;Fig 1.tif

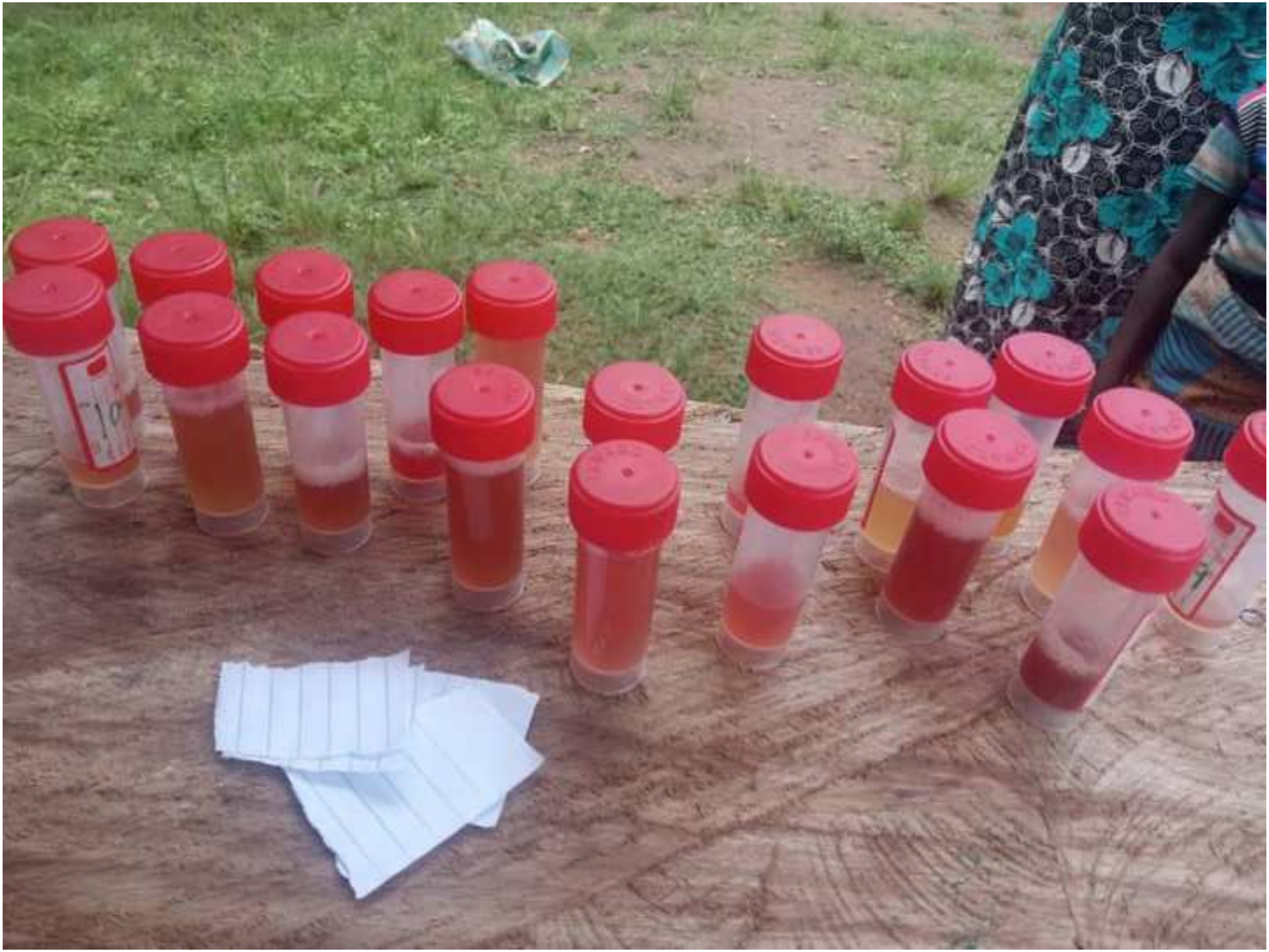

Figure 2 Click here to access/download;Figure;Untitled.tif

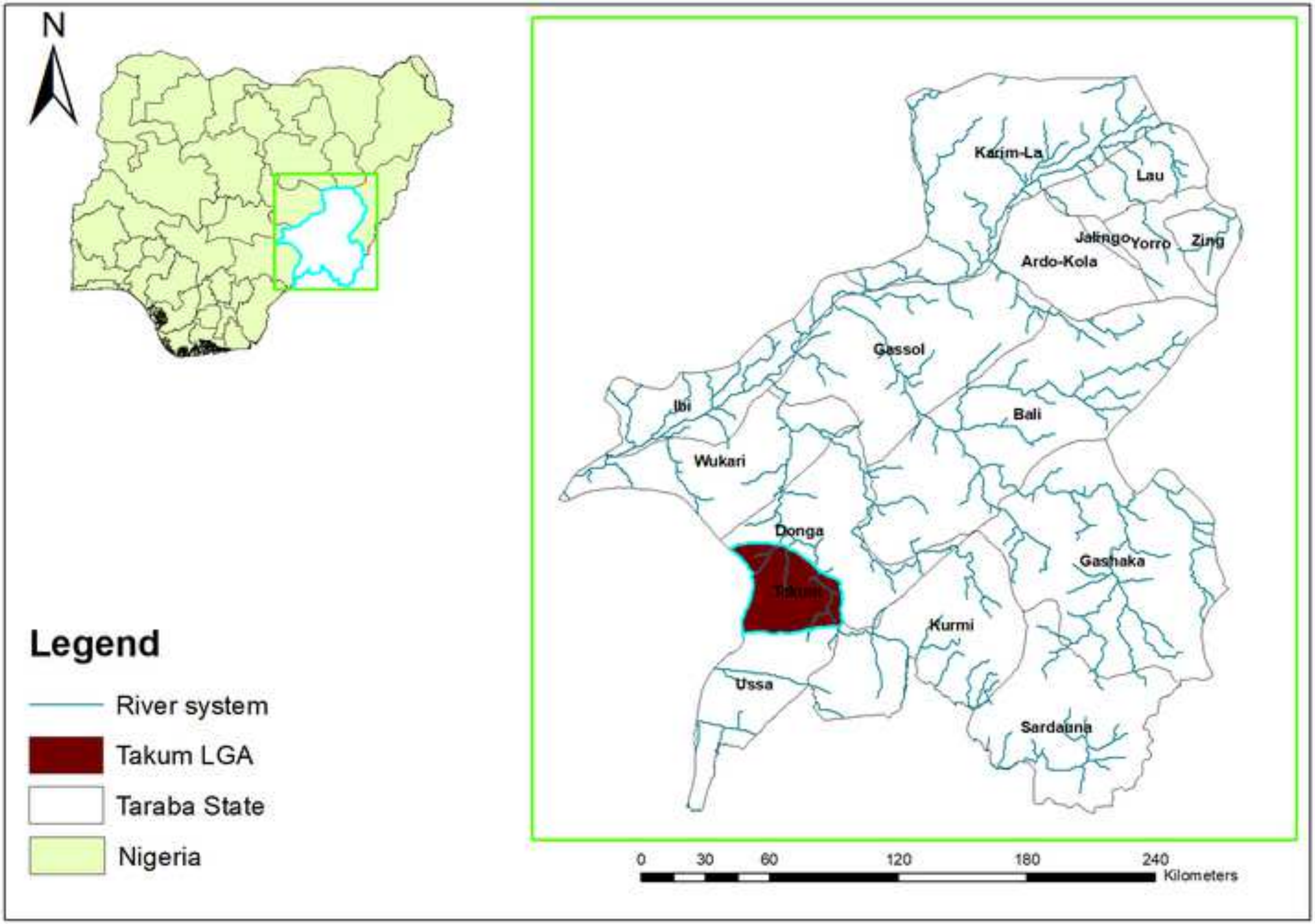

Figure 3

Click here to access/download;Figure;Fig 3.tif

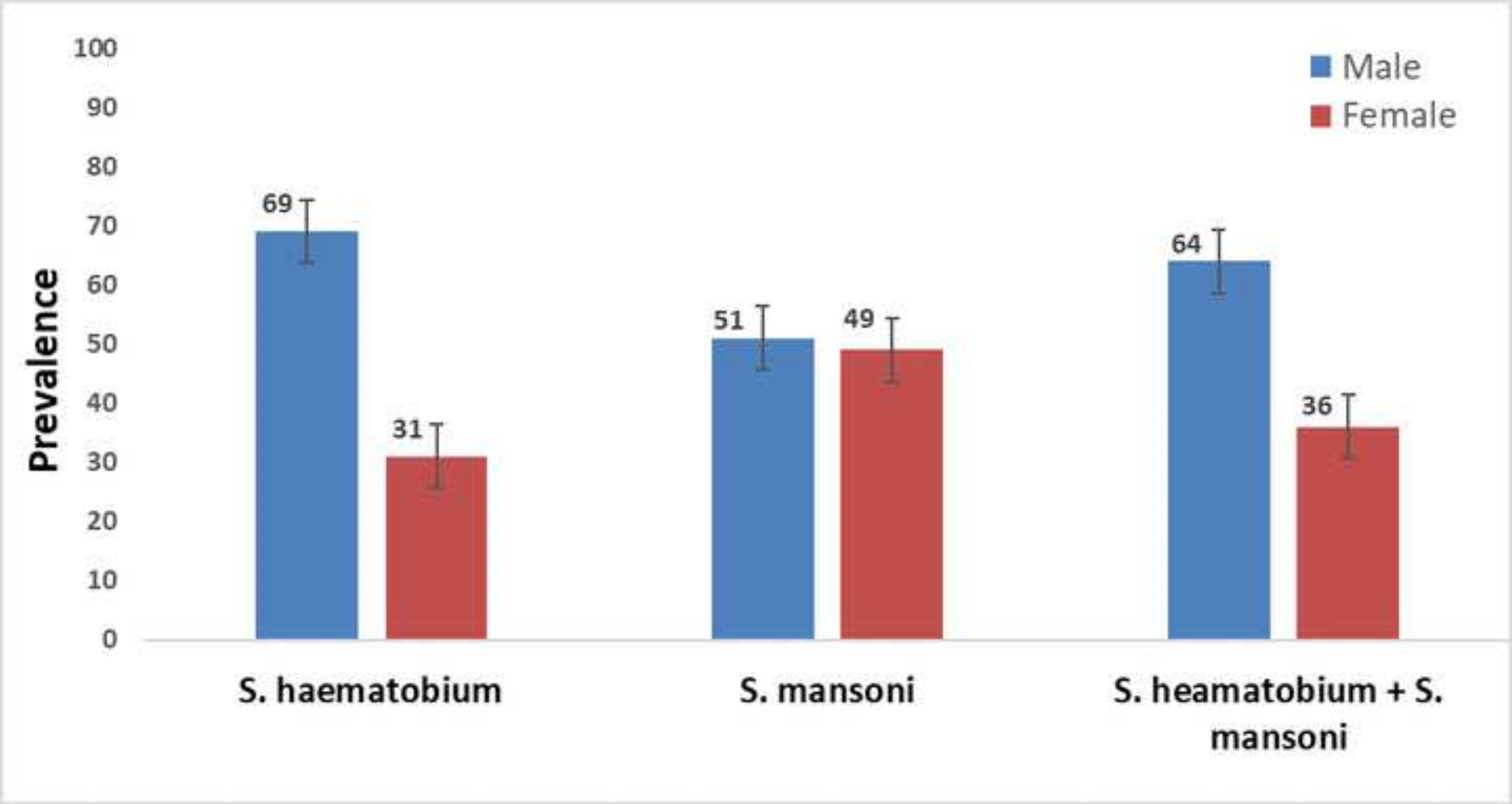

Figure 4

Click here to access/download;Figure;Fig 4.tif

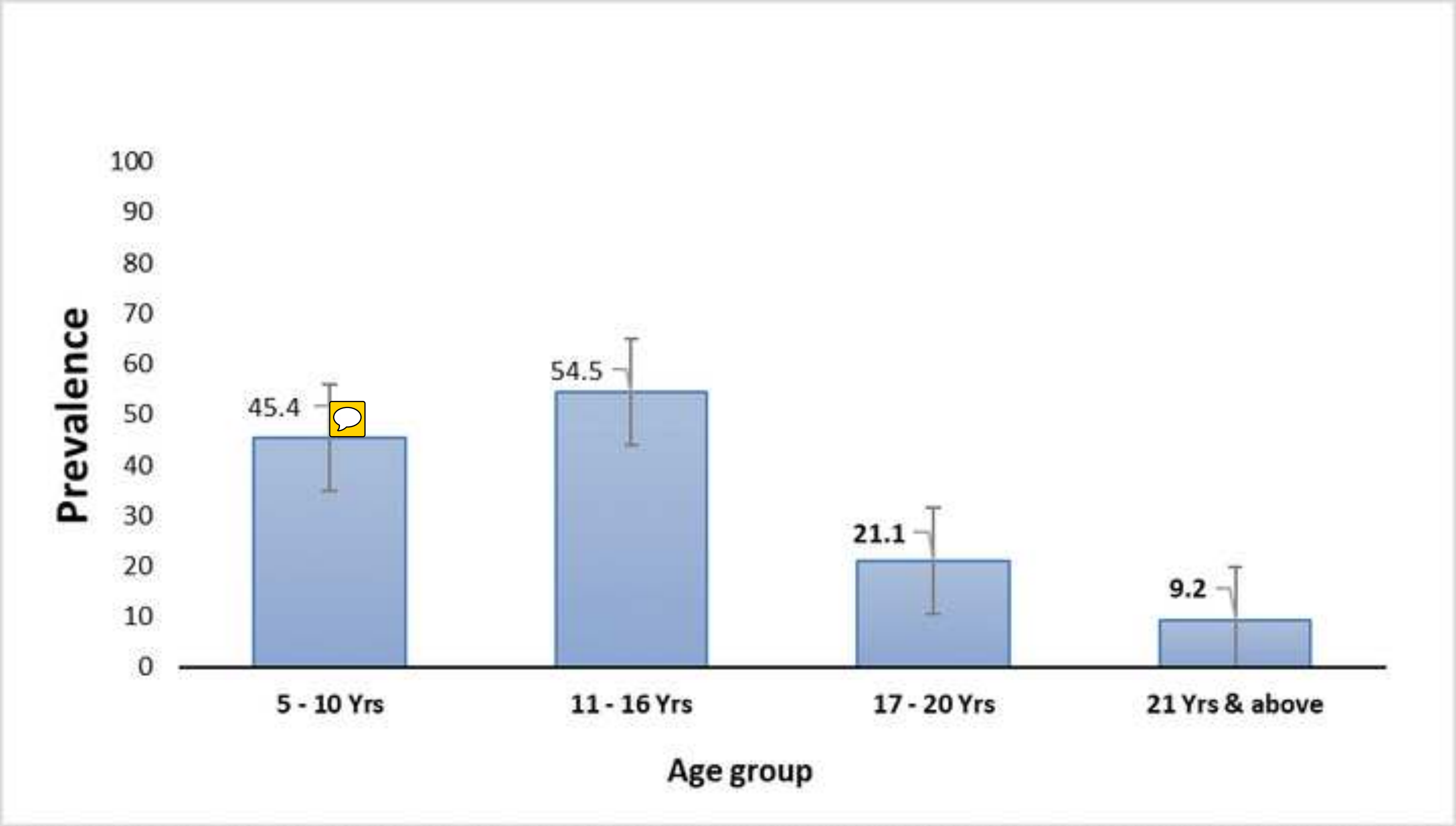

Supplement: Attachment — Submitted filename: PONE-D-21-30831_reviewer_new-1.pdf [file pone.0262524.s004.pdf]
