# Supplementary material for: Schistosomiasis outbreak during COVID-19 pandemic in Takum, Northeast Nigeria: Analysis of infection status and associated risk factors
Source: PLoS One. 2022 Jan 21;17(1):e0262524. doi: 10.1371/journal.pone.0262524 (PMC8782311; doi:10.1371/journal.pone.0262524)
Supplement: Attachment — Submitted filename: REVIEWERS REPORT.docx [file pone.0262524.s003.docx]

**REVIEWER’S REPORT.**

The paper is addressing a key issue in public health i.e An outbreak of schistosomiasis in Takum LGA in Northeastern Nigeria. However, the paper has some shortfalls and I suggest you consider the following:

1. The methodology needs some clarification, administration of drugs to people was larger than the sample surveyed. The use of error bar seems misleading and needed to be re-address
2. Though the paper was well presented, however, some aspect needed for follow a chronological order so as to have free flow when reading the paper if accepted for publication.
3. The gap which was identified needed to be filled through right discussion on good implementation research
4. The discussion needs to be aligned to the key results of the study and avoid reference to stuff that was note presented in the results.
5. The present manuscript requires editing for grammar, spelling errors and further concision.
