# Supplementary material for: Schistosomiasis outbreak during COVID-19 pandemic in Takum, Northeast Nigeria: Analysis of infection status and associated risk factors
Source: PLoS One. 2022 Jan 21;17(1):e0262524. doi: 10.1371/journal.pone.0262524 (PMC8782311; doi:10.1371/journal.pone.0262524)
Supplement: Attachment — Submitted filename: Response to Reviewer 2 comments.docx [file pone.0262524.s005.docx]

**REVIEWER’S REPORT.**

**Summary**

The paper is addressing a key issue in public health i.e., An outbreak of schistosomiasis in Takum LGA in Northeastern Nigeria. However, the paper has some shortfalls and I suggest you consider the following:

C1: The methodology needs some clarification, administration of drugs to people was larger than the sample surveyed. The use of error bar seems misleading and needed to be re-address

R1: Thank you very much for the valuable comments. Foremost, we explained within the manuscript that the epidemiological team responded to the outbreak by implementing mass administration of praziquantel to all eligible persons in the communities through the usual door-door preventive chemotherapy approach as recommended by the WHO. This approach doesn’t require prior diagnosis of infection before administration of tablets. Prior to the COVID-19 pandemic, this treatment is planned biennially (twice in a year) by the LGA NTD unit, but was ceased due to the COVID-19 pandemic. However, because of the outbreak reported, it became imperative to resume treatment in this area, which coincided with the time the epidemiological investigation was conducted. For this reason, the number of persons treated were larger in number than those surveyed in the study.

Secondly, for the figures, we used standard deviation to illustrate the margin between the individual infection data. The authors prefer to use this metric in place of standard error which measures the accuracy of estimations.

C2: Though the paper was well presented, however, some aspect needed for follow a chronological order so as to have free flow when reading the paper if accepted for publication.

R2: Thank you very much for this valuable comment. We have revised this section to allow more thoughtful flow. We really appreciate the reviewer for pointing this out. We have revised several parts of the manuscripts to allow more chronological flow.

C3: The gap which was identified needed to be filled through right discussion on good implementation research

R3: Under the recommendation paragraphs and conclusion remarks, we have re-emphasized how to address the gaps that was identified. This comment was also addressed under the more specific comments raised by the reviewer (C25)

C4: The discussion needs to be aligned to the key results of the study and avoid reference to stuff that was note presented in the results.

R4: Thank you for this comment. We really appreciate your inputs. We have also addressed this comment under the more specific comments raised by the reviewer (C22 and C24)

C5: The present manuscript requires editing for grammar, spelling errors and further concision.

R5: We take this opportunity to appreciate the reviewer for the invaluable comments. We have worked through the manuscript and have corrected the grammar, spelling errors and deleted some unnecessary sentences

**Other comments**

C1: Line 2. Abstract, kindly recast, there cannot be an outbreak of haematuria, it is just one of the symptoms of schistosomiasis

R1: Thank you very much for this valuable comment. We think PloS ONE goes with the American style.

C2; Page 2, keyword on hematuria, Please, be consistent with the choice of your English, it's either you go with American style of British.

R2: Thank you very much for this comment.

C3: Page 10, Line 3 under Introduction, "one of the major and most.." please, kindly recast, it is seems ambiguous!

R3: Thank you very much for this comment. We have revised it to “one of the most”

C4; Page 11, Line 1, For haematuria as a sign of the infection, most times, visible blood is noticed, you know?

R4: Thank you very much for this comment. We have revised the word symptom to sign. We thank you for this valuable addition.

C5: Page 22, Paragraph 2, Line 1, what is the unit of your age here?

R5: Thank you very much for this comment. We have revised it to “Children under 15 years of age….

C6: Page 22, Paragraph 2, Line 8 "LGA" is appearing for the first here, yet, you had only "government areas" here.?????

R6: Thank you very much for this comment. We have revised it to “local government areas….

C7: Page 22, Paragraph 2, Line 13, I would suggest, you allow your information to flow in a chronological order.

R7: Thank you very much for this valuable comment. We have revised it to allow more thoughtful flow. Please see the revised text below:

Nigeria is one of the schistosomiasis endemic countries in Africa [1], with 36 states and 774 local government areas (LGAs). About 708 LGAs had been mapped by the Federal Ministry of Health (FMoH), with 608 of them being endemic [16]. Since 2009, treatment with praziquantel commenced in 27 states with the support of WHO, UNICEF and partner organizations such as Mission to save the helpless (MITOSATH), Sightsavers, AMEN foundation among others [16]. Taraba, was among the states in mapped for schistosomiasis in 2010 and subsequently in 2014 [16,17]. The state is located in the northeastern region of the country, and has 16 LGAs.

C8: Page 12, Line 3, how many Takum community/LGA do you have in that LGA???

R8: Thank you very much for this valuable comment. We have highlighted this and revised accordingly. We have 2 different communities with the name Takum A and Takum B. Thanks for pointing this out.

C9: : Page 12, Line 7, ????

R9: Thank you very much for this valuable comment. We have highlighted this and revised accordingly. We have replaced the word hematuria with schistosomiasis

C10: Page 12, Line 11, Kindly correct your error here!!!!!

R10: Thank you very much for pointing out this error. We have deleted the repeated word.

C11: Page 13, under study area, Are you the first to carry out an investigation in that LGA? If your answer is NO. kindly, provide the reference for the coordinate provided here!!!!

R11: Thank you very much for this. We have provided a reference to support the description of the study area.

C12: Page 13, Line 4, under study design, kindly recast, this sentence is ambiguous!!!!!!!

R12: Thank you very much for pointing this out. We have reworked the sentence to provide more clarity

Please see below

The five study communities (Barkin lissa, Birama, Gamga, Shibong and Takpa) were randomly selected using the paper ballot approach out of the eleven communities.

C13; Page 13, Line 9, under study design, Again, you sentences should be in chronological order. I didn't find these statement easy to read.

R13: Thank you very much for pointing this out. We have reworked the sentence to provide more clarity

Please see below

Preliminary advocacy visits were made to the NTD control unit closer to the selected communities (i.e., ward level), prior to the epidemiological investigation

C14; Page 13, Line 3, under sample size, You invited all members of the community for the study, yet, you stated before that you had a method of choosing who to participate????? OR

does the study involved a purposive method????

R14: Thank you very much for your very valuable comment, which has improved the quality of our manuscript. We have now provided very explicit details on how the sample-size were estimated and how the participants were recruited.

Please find below

***Sample size determination and selection of study participants***

As an initial step, the total population of school-aged children and adults in the communities were extracted from the 2020 village census register obtained from the NTDs control department in the LGA. A total of 6,012 persons comprising 2531 children and 3481 adults were enumerated. As a further step, a sample-size was determined using the formula; $\boldsymbol{n}_{\boldsymbol{s}}\boldsymbol{=}\frac{\boldsymbol{n}}{\boldsymbol{1+}\left( \boldsymbol{n/N} \right)}$ and $\boldsymbol{n=}\frac{\boldsymbol{z}^{\boldsymbol{2}}\boldsymbol{p(1-p)}}{\boldsymbol{d}^{\boldsymbol{2}}}$ , as described by Lwanga et al [18], where n_s_ is the required sample size and N is the target population. We assumed a prevalence (p) of 50% since there are no previous baseline data on schistosomiasis in the five communities, a relative precision (d) of 5% and a confidence level of 95% which corresponds to z score 1.96. The minimum sample size determined, therefore, was 362 i.e., an average of 72 persons per community. However, the recruitment of participants extended beyond the estimated sample size, considering the aim of identifying factors associated with the outbreak.

For the selection of study participants, we employed a total sampling methodology, following the method previously described by [19]. Community sensitization and advocacy visits were made to the community leaders and other stakeholders. This was followed by mobilization of eligible community members to participate in the study using town announcers. Only residents of the community, who are above age of 5 years, can provide consent or assent, and has lived within the community in the last 3 months were recruited into the study. Enrollment of participants and collection of samples took place at a central point in the community, provided by the community leader. This point has a secluded space for administering study questionnaires and sorting of samples before transporting them to the laboratory. The number of consenting participants varied across the communities, hence giving roughly an unequal number of persons recruited.

C15: Page 16, Line 5, Giving of these incentives looks as if you used those items to entice them for the study which it seems not ethical, if you had provided this information to your ethical review committee, maybe, it may have been spotted and provide necessary advice!!!!!!!!

R15: Thank you very much for your comments. These incentives were not used to entice the participants, as they were given even to those that refused to participate in the study but visited the collection point. This information was provided in our letter of request, it was highlighted that the immediate benefits from the study would be provision of treatment to all eligible members of the communities, and incentives such as bar soaps to reinforce positive hygiene behaviors and bisquits to stimulate community integration especially during a tensed situation which the pandemic has presented. As such we guided the time of introduction of the incentives, ensuring it is non-coercive and doesn’t influence their participation in the research

Thank you very much for this valuable comment, and we have added some lines to support the sentence.

Please see below

All eligible and non-eligible participants were treated with 400mg/kg of praziquantel as an immediate benefit of the research investigation. In addition to this, incentives such as bar soaps and bisquits were given to reinforce positive hygiene behaviors and stimulate community integration during a tensed situation which the pandemic has presented. These incentives were provided in a non-coercive manner, ensuring they do not influence the decision of the community members to participate or decline participation in the research

C16: Page 16, Line 12, under parasitological assessment, Did you divide the infection into categories????

R16: Yes the infection were divided into categories. One category for S. haematobium and the other for S. mansoni. Thank you for your comment.

C17: Page 16, Line 2, under treatment, The use of your "all" is confusing. do you mean all infected individual or all communities members who came for the test?

R17: Thank you very much for your comment. Yes we treated all eligible members of the community, irrespective of their participation in the study. This followed the routine guidelines for implementing MDA in the country. Door to door visitation was made and praziquantel was administered to every member of the community.

We have improved our text to highlight this.

C18: Page 18, under results, Line 4, What is the basis for checking for the significant difference here????

R18: Thank you for your comments here. Really there is no major basis here other than the fact that it gives prior information that the distribution of participants varied significantly by gender across the communities. Which is quite typical of most cross-sectional epidemiological studies. But we don’t think it add much value to the paper, or reduces it. But we would love to retain them if the reviewers don’t mind.

C19: Page 20, title,????

R19: Thank you very much for your comment. We really appreciate it

C20: Page 20, Line 1, under treatment data, 432 participated in the investigation, you treated 3,580 people. How do you explain the rationale for this action? Did you just treat people without conducting an appropriate test?

R20: Thank you very much for the valuable comments. Foremost, we explained within the manuscript that the epidemiological team responded to the outbreak by implementing mass administration of praziquantel to all eligible persons in the communities through the usual door-door preventive chemotherapy approach as recommended by the WHO. This approach doesn’t require prior diagnosis of infection before administration of tablets. Prior to the COVID-19 pandemic, this treatment is planned annually (once in a year) by the LGA NTD unit, but was ceased due to the COVID-19 pandemic. However, because of the outbreak reported, it became imperative to resume treatment in this area, which coincided with the time the epidemiological investigation was conducted. For this reason, the number of persons treated were larger in number than those surveyed in the study.

C21: Page 20, line 3, under treatment data, Please what was the rationale for this????

R21: Thank you very much for your comments. The response to this comment has been provided above. We really appreciate your contributions.

C22: Page 21, Line 2, under discussion, This statement is not convincing enough!!!!

R22: Thank you very much for pointing this to us. This really improved the flow of our text. We have modified the text, and added a reference to support our statement here.

Please see below

Since 2014, Takum LGA has benefitted from three biennial rounds of MDA, which targeted 75% of its school-aged children. In these years, the therapeutic coverage were optimal, surpassing the 75% national targets, as such, the outbreak of urogenital schistosomiasis was unexpected [17].

C23: Page 21, Paragraph 2, line 1, ????????

R23: Thank you very much, we have improved the text, and changed the word from hematuria to schistosomiasis.

C24: Page 21, Paragraph 2, Line 4, You are discussing what you did not show in your result, why???

R24: Thank you very much for this observation. We have provided the results been presented here on Table 2. Now with your comments we found it necessary to improve the table and also include a footnote with thte table to describe the categories of endemicity based on prevalence value. We take this time to thank you once more for the valuable comments.

Please see below

**Categories of Endemicity**:

^a^Low endemicity when prevalence is between 1-9.9%;

^b^Moderate endemicity when prevalence is between 10-49.9%

^c^High endemicity when prevalence is above 50%)

C25: Page 23, Line 1, under conclusion, since you identified a gap in the implementation of drug delivery by FMoH and other for the treatment of the disease, I thought you will focus more on how to address right implementation of drug delivery but little or nothing is said about it in this conclusion and recommendation.

R25: Thank you very much once again for this very valuable comment. We have now adjusted the recommendation and conclusion lines, to buttress these points. Thanks.

Please see below

Under recommendations:

It is therefore imperative to consider; (1) investments in effort targeted at reclassifying the LGA as highly endemic, and adjusting the MDA thresholds from the biennial cycle to an annual cycle

Under conclusions:

Until now, Takum was classified to be a low endemic LGA and had been receiving biennial treatment. This outbreak and our prevalence reports highlight the need to re-classify the LGA as highly endemic, and adjust the MDA thresholds from the biennial cycle to an annual cycle

C26: Figure 2: Your error bar looks misleading, if you use standard error data for your error bar, I don't think you will have this high error bar for your data!!!!

R26: Thank you so much for all your wonderful comments, which were really helpful. For the figures, we used standard deviation to illustrate the margin between the individual infection data. The authors prefer to use this metric in place of standard error which measures the accuracy of estimations.
