# Supplementary material for: Schistosomiasis outbreak during COVID-19 pandemic in Takum, Northeast Nigeria: Analysis of infection status and associated risk factors
Source: PLoS One. 2022 Jan 21;17(1):e0262524. doi: 10.1371/journal.pone.0262524 (PMC8782311; doi:10.1371/journal.pone.0262524)
Supplement: Attachment — Submitted filename: Response to Reviewer 2 Commnets.docx [file pone.0262524.s006.docx]

Reviewer #2

C1:  They did a good job by updating the manuscript, I really commend them. However, I will encourage the authors to look into the error bars in figure 3 and 4, the error bars are too high giving the impression that there are so meny errors assosiated with the data collected. Thank you.

R1: Thank you very much for your very valuable comment, which has improved the quality of our manuscript. We agree completely with you. We have re-worked the error-bars using percentage error bars, because both SE and SD error bars were too high after trying them. We have provided new figures showing these new error bars. We take this opportunity to appreciate the reviewers and editor for the great efforts on our manuscript.
